# 12th Brazilian Congress of Epidemiology: innovative solutions for contemporary health challenges

**DOI:** 10.1590/0102-311XEN230224

**Published:** 2025-01-27

**Authors:** Claudia Leite de Moraes, Enirtes Caetano Prates Melo, Tânia Maria de Araújo

**Affiliations:** 1 Instituto de Medicina Social Hesio Cordeiro, Universidade do Estado do Rio de Janeiro, Rio de Janeiro, Brasil.; 2 Programa de Pós-graduação em Saúde da Família, Universidade Estácio de Sá/IDOMED, Rio de Janeiro, Brasil.; 3 Escola Nacional de Saúde Pública Sergio Arouca, Fundação Oswaldo Cruz, Rio de Janeiro, Brasil.; 4 Universidade Estadual de Feira de Santana, Feira de Santana, Brasil.

*The Epidemiology and the Complexity of Health Challenges* was the central theme of the 12th Brazilian Congress of Epidemiology (12th Epi), which was held on September 24-27, 2024, in Rio de Janeiro, Brazil. Complexity implies that systems, issues, and phenomena cannot be understood only through the isolated analysis of their parts. Therefore, one must approach them in an integrative manner in order to be able to capture multi-contextual realities ^1^. Thus, the conception of the Congress was anchored on the idea that reality must be understood as an interconnected whole that embraces the uncertainty and interdependence of systems and is based on the concept that interactions between parts results in new properties. Such properties require − in the search for adequate and effective responses − a dynamic and adaptable view that is able to handle paradoxes and transformations. Considering this complexity, the theme enabled discussions on multiple and diverse scenarios and challenges of Brazilian healthcare settings, highlighting the ability of Brazilian Epidemiology to create innovative and inclusive strategies based on democratic, plural, and broadly participatory processes to face such challenges.

The main challenges addressed in the congress included climate change, which refers to changes in climate patterns, impacting environmental dynamics and directly influencing the occurrence, frequency, and magnitude of extreme phenomena, such as droughts, floods, and heat waves ^2^. These events, in turn, impact the proliferation of vectors and increase the occurrence of diseases in unforeseen periods or in previously unaffected areas. Such scenarios require the development of new abilities to respond to, predict, and anticipate unusual events, in addition to improving the approach to the complexities associated with different morbidity and mortality profiles. These issues are aggravated by the enormous social inequities that, by overlapping vulnerabilities, disproportionately affect the poorest, black, and Indigenous populations.

In response to these challenges, 12th Epi incorporated a new thematic axis into its structure: *Public Health Emergencies, Global Climate Issues, and Production Models*. It addressed climate change, its interactions with human labor and production processes, and its effects on health and public health emergencies. The axis of *Interfaces with Society: Information, Education, and Communication in Health* was also emphasized, which discussed the essential role of disseminating and translating knowledge to support science and actions based on scientific evidence. The historical commitments of Brazilian epidemiology continued to guide activities under the axes *Theoretical and Methodological Challenges and Advances*, *Health Surveillance: Innovation, Information, and Action*, *Challenges in Epidemiology Training*, *Sustainability, Dissemination, and Integrity in Research*, *Innovative Approaches in Epidemiological Studies Focusing on Health Issues and Specific Population Groups*, and *Social Determination of Health-Disease Processes in Contexts of Expanded Vulnerability*. The congress was structured around eight contemporary thematic axes in the field, exploring the frontiers of knowledge and identifying future possibilities of research and public policy to cover this wide range of interests.

The many challenges related to the health issues observed in these thematic areas include the relevance and opportunity to advance the discussion and propose actions. The inequalities produced by a greedy neoliberal economic model that concentrates wealth − as well as their effects on health − emerged as central themes and cross-cutting agendas throughout the event. Initiatives that address hunger and poverty were also highlighted. Social determinants of health were widely discussed in conferences, lectures, and other activities, reinforcing that the production of knowledge on these inequities and their impacts on health is essential to strengthen actions that promote equitable access to public policies and health services. Furthermore, research and experiences to reduce regional, economic, gender and race/color inequalities were highlighted, aiming at consolidating a truly Public Health perspective.

Rio de Janeiro recently became the center of global attention when it hosted the 19th meeting of the G20 Summitt. During the preparatory activities, strategic priorities for the global agenda were discussed, including prevention, readiness, and response to pandemics, with an emphasis on strengthening local and regional production of medicines, vaccines, and inputs. Other aspects included digital health, aiming at expanding telehealth, integrating national health systems, and analyzing data to optimize services. Health equity and the impacts of climate change on health were also discussed, reinforcing the relevance of these topics in building more resilient and sustainable health systems. These priorities were also valued in our program.

Health crises, as evidenced by the COVID-19 pandemic, highlight the urgent need to develop rapid response strategies that include robust monitoring and surveillance systems, data integration, and engagement with local communities. The initiatives of several groups of Brazilian epidemiologists, widely disseminated in 12th Epi activities, have made significant progress in this sense and helped strengthen the ability to handle public health emergencies. In a scenario in which vaccine hesitancy is aggravated by misinformation, the role of epidemiology in strengthening confidence in vaccines and other effective public health interventions was reinforced at the congress. The use of digital innovations − such as Big Data analysis and artificial intelligence tools − was also highlighted, exploring their potential and the challenges related to data privacy and the responsible use of such information. The burden of chronic diseases and mental health problems were also widely discussed in several activities, providing valuable reflections on the innovations needed in health surveillance, prevention strategies, lines of care, and other actions that address these challenges in the Brazilian Unified National Health System (SUS, acronym in Portuguese).

In this scenario, violence, deeply rooted in our slave and sexist culture, was also at the center of the debate. Racism, institutional violence, urban violence, gender-based violence, abuse against children and adolescents, self-harm, and suicide were some of the debated topics. It was emphasized that violence not only takes lives, but also causes serious harm to the health of survivors, their families, and society as a whole. Although it affects a large part of the Brazilian population, its consequences are especially devastating for the most vulnerable groups, such as women, children, adolescents, and young poor black people who live in the outskirts of cities.

Since the 1st Brazilian Congress of Epidemiology, held in 1990 in Campinas (São Paulo State), we have seen how the diversity of topics, the exchange of experiences, learning, meetings, and the strengthening of the field have been strong characteristics of these events. The same elements contributed to the great success of the 12th Epi, which had around 3,400 attendees from all regions of Brazil and other countries. The event was more than just a meeting of researchers and health professionals; it became a symbol of Brazilian Public Health Association’s (Abrasco, acronym in Portuguese) commitment, especially of its Epidemiology Committee, to democracy, strengthening public health, defending the SUS, valuing diversity, and reducing inequalities. The various actions implemented in this regard throughout the organization of the congress included the commitment of the Scientific Committee and the Local Organization to promoting diversity in its multiple dimensions − thematic, institutional, generational, regional, gender, and race/ethnicity/color − in the composition of roundtables, lectures, and conferences. In addition, affirmative actions were adopted to increase the participation of vulnerable populations and volunteer supervisors, including exemption from registration fees and the provision of meals. These initiatives, guaranteed by the Local Organization Committee, significantly enriched the discussions during the event.

The congress schedule program was broad and varied. The Pre-Congress, held at State University of Rio de Janeiro (UERJ, acronym in Portuguese), offered 48 activities distributed among 25 courses, 21 workshops, and two meetings. 12th Epi, in turn, offered seven conferences, eight lectures and 62 roundtables, which involved 234 guests and 94 moderators. Most activities were developed with the active participation of the community of epidemiologists from all over the country, through an open public call for courses, workshops, roundtables, and lectures. The activities of coordinated communications, dialogue posters, and electronic posters also reflected the broad and active participation of the community, with works developed at different levels of training and practice from all over the country. A total of 3,380 works were submitted, of which 2,802 were research reports (82.9%) and 579 were experience reports (17.1%). The large number of approved abstracts resulted in 65 coordinated communication sessions, including 312 papers, 60 dialogue poster sessions with the presentations of 314 papers. Among the coordinated communications, 15 papers were had honorable mentions. The most frequently discussed topics were: communicable diseases; chronic noncommunicable diseases; epidemiological surveillance and health surveillance; nutritional epidemiology; child and adolescent health; evaluation of medical-health systems, policies, programs, services, and technologies; adult health/aging; workers’ health; epidemiology in specific population subgroups; and accidents and violence, among others. The number of papers on each of these topics will be available in the congress annals, which will be published soon by Abrasco.

In addition to the rich scientific program, we held three Intergenerational Meetings to foster dialogue between the generations that contributed to the construction and consolidation of Epidemiology in Brazil and young researchers. These meetings highlighted the importance of exchanging experiences as an essential tool to promote equity in health. Another innovative initiative of this edition of the congress was the creation of a space for the debate of the main theoretical, conceptual, and methodological challenges in the development of large epidemiological studies in Brazil. Covering a wide range of study topics and areas of activity, this unprecedented activity had 16 sessions with 38 guests and attracted more than 100 participants. Complementing the program, the Scientific Committee, in partnership with Abrasco Livros and Editora Fiocruz, organized the launch of 16 books on various thematic areas. This initiative represented an important stimulus to scientific production and the advance of knowledge in the field, with potential to inspire new studies and transformative actions.

Also, in a special session of the congress, the V Master Plan for the Development of Epidemiology in Brazil (2025-2029) was launched. This milestone signaled the resumption of 5-year master plans for Epidemiology in the country, a practice that began in 1989 but was interrupted in 2005. Developed in an extensive process of collective construction that began in 2022, the V Master Plan defines the next steps of the development and future of Epidemiology in Brazil. Its guidelines define advances in the training of professionals, research, data integration, and the implementation of more effective public policies in order to promote health, control diseases, and reduce social, regional, gender, racial, and ethnic inequalities.

All these activities reflected the strength, breadth, and diversity of this field. Creating such a rich congress was only possible thanks to meticulous collective work supported by partnerships, solidarity, common goals, and a deep commitment to public health and epidemiology - all combined with tireless dedication. The Scientific Committee, with its 36 members, played a central role, gathering efforts of the Abrasco epidemiology committee, researchers from different institutions, and professionals from various departments of the Department of Health and Environmental Surveillance of the Ministry of Health. The Local Committee had the participation of 31 members from the institutions that promoted the Congress - UERJ, Oswaldo Cruz Foundation (FIOCRUZ, acronym in Portuguese), Federal University of Rio de Janeiro (UFRJ, acronym in Portuguese), Fluminense Federal University (UFF, acronym in Portuguese), and Institute for Medical Education (IDOMED, acronym in Portuguese). The Abrasco Secretariat was committed and dedicated to supporting the actions and the Presidency of the congress actively participated in all these actions. 12th Epi, in all its magnitude, was the result of this collective strength and the joint effort of all everyone involved.

We would also like to highlight the progress made towards gender equity in Brazilian epidemiology. Women had coordination roles in the organization and program development: there were two female presidents of the Congress, in addition to two female honorary presidents, one female president of the Scientific Committee, and two female coordinators of the Local Committee. The congress also recognized researchers with outstanding contributions to the development of epidemiology in the country by honoring the following professors: Ligia Regina Franco Sansigolo Kerr (Federal University of Ceará), Antônio Augusto Moura da Silva (Federal University of Maranhão), Rosely Sichieri (UERJ), Célia Landmann Szwarcwald (FIOCRUZ), César Gomes Victora (Federal University of Pelotas), and José Eluf Neto (University of São Paulo, *in memoriam*).

The city of Rio de Janeiro, home of 12th Epi, is an emblematic symbol of the complexity and health challenges that characterize Brazil. For every Copacabana beach, there are threats from drug trafficking and militias. While emotions are celebrated at Maracanã, we deal with urban mobility issues; and next to Christ the Redeemer, we see vulnerable children. These contrasts reflect not only the reality of Rio de Janeiro, but also of Brazil as a whole, and reinforce the importance of holding meetings to discuss ways to change this reality.

With so many challenges, 12th Epi reaffirmed the transformative power of committed science that can generate solutions for building a more just and supportive future, that promotes social inclusion, respects diversity, and increases our understanding of the main health problems that affect the Brazilian population. In addition, it seeks partners from other countries with inspiring and innovative collaborations. This congress was more than a space to exchange knowledge; it was an opportunity to renew our commitment to strengthening science that fights against poverty, reduces inequalities, and guides policies that promote equity and social justice. 12th Epi was a firm response to the scientific denialism that marked the previous government administration. Science boycott and the dissemination of false information directly contributed to hundreds of thousands of deaths from COVID-19 and other diseases. In this context, the congress strengthened the relevance of evidence-based science and its essential role in defending life and public health.

Finally, we would like to highlight that throughout the entire process of congress organization, we sought to reinforce our commitment to democracy; the SUS; and the fight for a more just, less unequal, and discrimination-free society. The active participation of health professionals, researchers, and undergraduate and graduate students was the basis for the constructive and democratic debate on the themes of health complexity that mobilizes us. We, the organization committee of the 12th Epi, hope that the congress has inspired new ideas, strengthened commitments to a more just and supportive society, and renewed everyone’s energy to face the challenges of Public Health.

Motivated by so many problems to overcome, let’s move forward!

Long live science, long live the SUS, long live Abrasco and Brazilian Epidemiology!
