# Supplementary material for: 12th Brazilian Congress of Epidemiology: innovative solutions for contemporary health challenges
Source: Cad Saude Publica. 2025 Jan 27;41(1):e00230224. doi: 10.1590/0102-311XEN230224 (PMC11774329; doi:10.1590/0102-311XEN230224)
Supplement: Translation

# Translation: 12º Congresso Brasileiro de Epidemiologia: soluções inovadoras para os desafios sanitários contemporâneos

**DOI:** 10.1590/0102-311XPT230224

**Authors:** Claudia Leite de Moraes, Enirtes Caetano Prates Melo, Tânia Maria de Araújo

**Affiliations:** 1 Instituto de Medicina Social Hesio Cordeiro, Universidade do Estado do Rio de Janeiro, Rio de Janeiro, Brasil.; 2 Programa de Pós-graduação em Saúde da Família, Universidade Estácio de Sá/IDOMED, Rio de Janeiro, Brasil.; 3 Escola Nacional de Saúde Pública Sergio Arouca, Fundação Oswaldo Cruz, Rio de Janeiro, Brasil.; 4 Universidade Estadual de Feira de Santana, Feira de Santana, Brasil.

*A Epidemiologia e a Complexidade dos Desafios Sanitários* foi o tema central do 12º Congresso Brasileiro de Epidemiologia (12º Epi), que ocorreu entre 24 e 27 de setembro de 2024, no Rio de Janeiro, Brasil. O termo complexidade implica a ideia de que sistemas, problemas e fenômenos não podem ser entendidos apenas por meio da análise isolada das suas partes. Exigem, portanto, abordagens integradoras, capazes de captar realidades multicontextuais ^1^. Assim, a construção do Congresso ancorou-se na ideia de que a realidade deve ser compreendida como um todo interconectado, que reconhece a incerteza e a interdependência dos sistemas e parte da premissa de que as interações entre suas partes geram novas propriedades, o que exige, na busca por respostas adequadas e eficazes, uma visão dinâmica, adaptável e capaz de enfrentar paradoxos e transformações. Considerando essa complexidade, o tema escolhido permitiu iluminar os múltiplos e diversos cenários e desafios dos contextos sanitários brasileiros, destacando a capacidade da Epidemiologia brasileira de criar estratégias criativas, inclusivas e fundamentadas em processos democráticos, plurais e amplamente participativos para enfrentar esses desafios.

Entre os principais desafios abordados na agenda do congresso, destacaram-se as mudanças climáticas, que alteram eventos meteorológicos, afetam a dinâmica ambiental e influenciam diretamente a ocorrência, frequência e magnitude de fenômenos extremos, como secas, inundações e ondas de calor ^2^. Esses eventos, por sua vez, impactam na proliferação de vetores e ampliam a presença de doenças em períodos não previstos ou em áreas previamente não afetadas. Tais cenários demandam o desenvolvimento de novas capacidades para responder, prever e antecipar eventos inusitados, além de aprimoramento na abordagem das complexidades associadas a distintos perfis de morbimortalidade. Tais questões são agravadas pelas gigantescas iniquidades sociais que, ao sobrepor vulnerabilidades, afetam de maneira desproporcional as populações mais pobres, negras e indígenas.

Em resposta a esses desafios, o 12º Epi incorporou um novo eixo temático em sua estrutura, *Emergências em Saúde Pública, Questões Climáticas Globais e Modelos de Produção*. Esse eixo abordou as mudanças climáticas, suas interações com o trabalho humano e os processos produtivos e seus efeitos sobre a saúde e as emergências em saúde pública. Também ganhou ênfase o eixo *Interfaces com a Sociedade: Informação, Educação e Comunicação em Saúde*, que ressaltou o papel essencial da disseminação e tradução do conhecimento para a defesa da ciência e das ações amparadas em evidências científicas. Os compromissos históricos da epidemiologia brasileira continuaram a orientar as atividades com os eixos *Desafios e Avanços Teóricos e Metodológicos*, *Vigilância em Saúde: Inovação, Informação e Ação*, *Desafios na Formação em Epidemiologia*, *Sustentabilidade, Disseminação e Integridade em Pesquisa*, *Abordagens Inovadoras em Estudos Epidemiológicos com Foco em Problemas de Saúde e Grupos Populacionais Específicos* e *Determinação Social dos Processos Saúde-Doença em Contextos de Vulnerabilidade Ampliada*. Assim, a programação foi estruturada em torno dos oito eixos temáticos contemporâneos da área, explorando as fronteiras do conhecimento, identificando necessidades futuras de pesquisa e de políticas públicas capazes de atender a essa ampla constelação de interesses.

Entre os muitos desafios relacionados aos problemas de saúde abordados nesses eixos temáticos, destacou-se a relevância e a oportunidade de avançar na discussão e na proposição de ações para seu enfrentamento. As desigualdades produzidas por um modelo econômico neoliberal ganancioso e concentrador de riquezas, bem como seus efeitos na saúde emergiram como temas centrais e pautas transversais ao longo de todo o evento. Iniciativas voltadas para o enfrentamento da fome e da pobreza também ganharam destaque. Os determinantes sociais da saúde foram amplamente discutidos em conferências, palestras e outras atividades, reforçando a compreensão de que a produção de conhecimentos sobre essas iniquidades e seus impactos na saúde são essenciais para fortalecer ações que promovam acesso equitativo a políticas públicas e serviços de saúde. Adicionalmente, foram priorizadas pesquisas e experiências voltadas à redução das desigualdades regionais, econômicas, de gênero e de raça/cor, com o objetivo de consolidar uma perspectiva de saúde verdadeiramente coletiva.

Recentemente, o Rio de Janeiro tornou-se o centro das atenções globais ao sediar a 19ª Reunião da Cúpula do Grupo dos 20 (G20). Durante as atividades preparatórias, foram destacadas prioridades estratégicas para a agenda global, como a prevenção, preparação e resposta a pandemias, com ênfase no fortalecimento da produção local e regional de medicamentos, vacinas e insumos. Outros pontos incluíram a saúde digital, visando à ampliação da telessaúde, à integração dos sistemas nacionais de saúde e à análise de dados para otimizar os serviços. Questões de equidade em saúde e os impactos das mudanças climáticas na saúde também ganharam destaque, reforçando a relevância desses temas na construção de sistemas de saúde mais resilientes e sustentáveis. Essas prioridades foram igualmente valorizadas em nossa programação.

As crises sanitárias, como evidenciado pela pandemia de COVID-19, destacam a necessidade urgente de desenvolver estratégias de resposta rápida que incluam sistemas robustos de monitoramento e vigilância, integração de dados e engajamento das comunidades locais. As iniciativas de diversos grupos de epidemiologistas brasileiros, amplamente divulgadas nas atividades do 12º Epi, têm avançado significativamente nessa direção, contribuindo para o fortalecimento da capacidade de enfrentamento de emergências em saúde pública. Em um cenário em que a hesitação vacinal é agravada pela desinformação, reforçou-se no congresso o papel da epidemiologia no fortalecimento da confiança nas vacinas e em outras intervenções de saúde pública efetivas. O debate sobre inovações digitais − como a análise de *big data* e o uso de inteligência artificial (IA), também ocupou destaque, explorando tanto suas potencialidades, quanto os desafios relacionados à privacidade e ao uso responsável dos dados. Ademais, a carga de doenças crônicas e os agravos à saúde mental foram amplamente abordados em diversas atividades, proporcionando reflexões valiosas sobre as inovações necessárias na vigilância em saúde, nas estratégias de prevenção, nas linhas de cuidado e em outras ações voltadas ao enfrentamento desses desafios no Sistema Único de Saúde (SUS).

Ainda compondo esse cenário, estiveram no centro do debate as violências profundamente enraizadas em nossa cultura escravocrata e sexista. O racismo, a violência institucional, a violência urbana, a violência de gênero, os abusos contra crianças e adolescentes, as lesões autoprovocadas e o suicídio foram alguns dos temas discutidos. Reforçou-se que as violências não apenas ceifam vidas, mas também geram sérios prejuízos à saúde dos sobreviventes, de suas famílias e da sociedade como um todo. Embora atinjam grande parte da população brasileira, suas consequências são especialmente devastadoras para os grupos mais vulneráveis, como mulheres, crianças, adolescentes e jovens negros e pobres que vivem nas periferias.

Desde o 1º Congresso Brasileiro de Epidemiologia, realizado em 1990, em Campinas, São Paulo, temos testemunhado como a diversidade de temas, a troca de experiências, os aprendizados, os encontros e o fortalecimento da área têm sido marcas registradas dos eventos. Esses mesmos elementos contribuíram para o grande sucesso do 12º Epi. O congresso reuniu cerca de 3.400 participantes de todas as regiões do Brasil e de outros países. O evento foi mais do que um encontro de pesquisadores(as) e profissionais de saúde, este tornou-se um símbolo do compromisso da Associação Brasileira de Saúde Coletiva (Abrasco), em especial de sua Comissão de Epidemiologia, com a Democracia, o fortalecimento da saúde coletiva, a defesa do SUS, a valorização da diversidade e a redução das desigualdades. Entre as várias ações implementadas nesse sentido ao longo da organização do congresso, destacou-se o compromisso da Comissão Científica e da Organização Local com a promoção da diversidade em suas múltiplas dimensões − temática, institucional, geracional, regional, de gênero e de raça/etnia/cor − na composição de mesas-redondas, palestras e conferências. Além disso, ações afirmativas foram adotadas para ampliar a participação de populações vulnerabilizadas e monitores(as) voluntários(as), incluindo a isenção de taxas de inscrição e a oferta de refeições. Essas iniciativas, garantidas pela Comissão Organizadora Local, enriqueceram sobremaneira as discussões ao longo do evento.

A grade de programação do congresso foi ampla e variada. O Pré-Congresso, realizado na Universidade do Estado do Rio de Janeiro (UERJ), contou com 48 atividades, distribuídas entre 25 cursos, 21 oficinas e duas reuniões. O 12º Epi, por sua vez, contou com sete conferências, oito palestras e 62 mesas-redondas, que mobilizaram 234 convidados(as) e 94 moderadores(as). A maior parte das atividades foi construída com a participação ativa da comunidade de epidemiologistas de todo o país, por meio de uma chamada pública aberta para cursos, oficinas, mesas-redondas e palestras. As atividades de comunicações coordenadas, pôsteres dialogados e pôsteres eletrônicos também refletiram a ampla e ativa participação da comunidade, reunindo trabalhos desenvolvidos em diferentes níveis de formação e atuação, provenientes de todo o país; foram submetidos 3.380 trabalhos, dos quais 2.802 foram relatos de pesquisa (82,9%) e 579 de experiência (17,1%). O grande número de resumos aprovados resultou na realização de 65 sessões de comunicação coordenada, contemplando 312 trabalhos e 60 sessões de pôsteres dialogados com apresentação de 314 trabalhos. Entre as comunicações coordenadas, 15 trabalhos foram premiados com menção honrosa. Os temas mais abordados foram: doenças transmissíveis, doenças crônicas não-transmissíveis, vigilância epidemiológica e vigilância em saúde, epidemiologia nutricional, saúde da criança e do adolescente, avaliação de sistemas, políticas, programas, serviços e tecnologias médico-sanitárias, saúde do adulto/envelhecimento, saúde do trabalhador e da trabalhadora, epidemiologia em subgrupos populacionais específicos e acidentes e violências, entre outros. O número de trabalhos em cada um desses temas estará disponível nos anais do evento, que serão publicados em breve pela Abrasco.

Além da rica programação científica, promovemos três encontros intergeracionais, criados para fomentar o diálogo entre as gerações que contribuíram para a construção e consolidação da epidemiologia no Brasil e jovens pesquisadores(as). Esses encontros destacaram a importância da troca de experiências como ferramenta essencial para promover equidade na saúde. Outra iniciativa inovadora dessa edição do congresso foi a criação de um espaço dedicado ao debate dos principais desafios teóricos, conceituais e metodológicos enfrentados no desenvolvimento de grandes estudos epidemiológicos no Brasil. Abrangendo uma ampla gama de temas de estudo e áreas de atuação, essa atividade inédita contemplou 16 sessões, que reuniram 38 convidados(as) e atraíram mais de uma centena de congressistas. Complementando a programação, a comissão científica, em parceria com a Abrasco Livros e Editora Fiocruz, organizou o lançamento de 16 livros em diversos eixos temáticos. Essa iniciativa representou um importante estímulo à produção científica e ao avanço do conhecimento na área, com grande potencial para inspirar novas pesquisas e ações transformadoras.

O congresso também foi palco do lançamento do V Plano Diretor para o Desenvolvimento da Epidemiologia no Brasil (2025-2029), em uma sessão especial. Esse marco sinalizou a retomada dos planos diretores quinquenais para a Epidemiologia no país, uma prática iniciada em 1989, mas interrompida desde 2005. Elaborado ao longo de um extenso processo de construção coletiva iniciado em 2022, o V Plano Diretor propõe orientar os próximos passos no desenvolvimento e futuro da Epidemiologia no Brasil. Suas diretrizes visam avanços na formação de profissionais, na pesquisa, na integração de dados e na implementação de políticas públicas mais eficazes, com o objetivo de promover a saúde, controlar doenças e reduzir desigualdades sociais, regionais, de gênero, raciais e étnicas.

Todas estas atividades refletiram o vigor da área, sua amplitude e diversidade. A construção de um congresso com tamanha riqueza só foi possível graças a um trabalho coletivo minucioso, sustentado por parcerias, solidariedade, objetivos comuns e um profundo compromisso com a saúde coletiva e a epidemiologia − tudo isso aliado a uma dedicação incansável. A comissão científica, composta por 36 integrantes, desempenhou um papel central e reuniu esforços de pesquisadores de diferentes instituições, comissão de epidemiologia da Abrasco e de profissionais de diversos departamentos da Secretaria de Vigilância em Saúde e Ambiente do Ministério da Saúde. A comissão local, por sua vez, contou com a participação de 31 membros das instituições promotoras do Congresso − UERJ, Fundação Oswaldo Cruz (FIOCRUZ), Universidade Federal do Rio de Janeiro (UFRJ), Universidade Federal Fluminense (UFF) e Instituto de Educação Médica (IDOMED). A Secretaria da Abrasco esteve empenhada e dedicada ao apoio das ações e a presidência do congresso participou ativamente em todas essas frentes. O 12º Epi, em toda sua magnitude, foi o resultado dessa força coletiva e do empenho conjunto de todos os envolvidos.

Também gostaríamos de destacar os avanços em direção à equidade de gênero na epidemiologia brasileira. As mulheres assumiram os postos de coordenação na organização e programação: foram duas presidentas do Congresso, além de duas presidentas de honra, uma presidenta da comissão científica e duas coordenadoras da comissão local. O congresso também proporcionou um momento de reconhecimento aos pesquisadores e pesquisadoras com destacadas contribuições ao desenvolvimento da epidemiologia no país ao homenagear os(as) professores(as): Ligia Regina Franco Sansigolo Kerr (Universidade Federal do Ceará), Antônio Augusto Moura da Silva (Universidade Federal do Maranhão), Rosely Sichieri (UERJ), Célia Landmann Szwarcwald (FIOCRUZ), César Gomes Victora (Universidade Federal de Pelotas) e José Eluf Neto (Universidade de São Paulo, *in memoriam*).

A cidade do Rio de Janeiro, sede do 12º Epi, simboliza de forma emblemática a complexidade e os desafios sanitários que caracterizam nosso país. Para cada praia de Copacabana, enfrenta-se as ameaças do tráfico e das milícias; enquanto se celebram as emoções no Maracanã, lidamos com os problemas de mobilidade urbana; e ao lado do Cristo Redentor, convivemos com crianças em situação de vulnerabilidade. Esses contrastes refletem não apenas a realidade do Rio, mas também do Brasil como um todo, e reforçam a relevância de estarmos reunidos(as), discutindo caminhos para transformá-la.

Diante de tantos desafios, o 12º Epi reafirmou o poder transformador de uma ciência comprometida, capaz de gerar soluções para a construção de um futuro mais justo e solidário. Uma Ciência que promove a inclusão social, respeita a diversidade e aprofunda nosso entendimento sobre os principais problemas de saúde que afetam nossa população brasileira. Além disso, busca parceiros(as) de outras terras que tragam colaborações inspiradoras e inovadoras. Esse congresso foi mais do que um espaço para a troca de conhecimento, mostrou-se uma oportunidade de renovar nosso compromisso com o fortalecimento de uma ciência que combate à pobreza, reduz desigualdades e orienta políticas que visem à equidade e à justiça social. O 12º Epi foi uma resposta firme ao negacionismo científico que marcou o governo anterior. O boicote à ciência e o incentivo à disseminação de informações falsas contribuíram diretamente para centenas de milhares de mortes por COVID-19 e outras doenças. Nesse contexto, o congresso reafirmou a relevância da ciência baseada em evidências e seu papel essencial na defesa da vida e da saúde pública.

Por fim, gostaríamos de reafirmar que ao longo de todo o processo de construção do evento, buscou-se reforçar nosso compromisso com a Democracia, o SUS, a luta por uma sociedade mais justa, menos desigual e livre de discriminações. A participação plena de profissionais de saúde, pesquisadores(as), discentes de graduação e pós-graduação foram a base para o debate construtivo e democrático sobre os temas que compõem a complexidade sanitária que tanto nos mobiliza. Nós da comissão organizadora do 12º Epi esperamos que o congresso tenha inspirado novas ideias, fortalecido os compromissos com uma sociedade mais justa e solidária e renovado as energias para enfrentar os desafios da Saúde Coletiva.

Motivados por tantos problemas a superar, vamos em frente!

Viva a Ciência, viva o SUS, viva a Abrasco e a Epidemiologia brasileira!

# Translation: 12.º Congreso Brasileño de Epidemiología: soluciones innovadoras para los desafíos sanitarios contemporáneos

**DOI:** 10.1590/0102-311XES230224

**Authors:** Claudia Leite de Moraes, Enirtes Caetano Prates Melo, Tânia Maria de Araújo

**Affiliations:** 1 Instituto de Medicina Social Hesio Cordeiro, Universidade do Estado do Rio de Janeiro, Rio de Janeiro, Brasil.; 2 Programa de Pós-graduação em Saúde da Família, Universidade Estácio de Sá/IDOMED, Rio de Janeiro, Brasil.; 3 Escola Nacional de Saúde Pública Sergio Arouca, Fundação Oswaldo Cruz, Rio de Janeiro, Brasil.; 4 Universidade Estadual de Feira de Santana, Feira de Santana, Brasil.

*La Epidemiología y la Complejidad de los Desafíos de Salud* fue el tema central del 12.º Congreso Brasileño de Epidemiología (12.º Epi), que tuvo lugar entre el 24 y el 27 de septiembre del 2024, en Río de Janeiro. El término complejidad implica la idea de que los sistemas, problemas y fenómenos no pueden entenderse simplemente mediante el análisis aislado de sus partes. Por lo tanto, requieren enfoques integradores, capaces de captar realidades multicontextuales ^1^. Así, la construcción del Congreso estuvo anclada en la idea de que la realidad debe entenderse como un todo interconectado, que reconoce la incertidumbre y la interdependencia de los sistemas y parte de la premisa de que las interacciones entre sus partes generan nuevas propiedades, lo que requiere, en la búsqueda por respuestas adecuadas y eficaces, una visión dinámica, adaptable y capaz de enfrentar paradojas y transformaciones. Considerando esta complejidad, el tema elegido permitió iluminar los múltiples y diversos escenarios y desafíos de los contextos sanitarios brasileños, resaltando la capacidad de la Epidemiología brasileña para crear estrategias creativas, inclusivas y basadas en procesos democráticos, plurales y ampliamente participativos para enfrentar esos desafíos.

Entre los principales desafíos abordados en la agenda del congreso, destacaron el cambio climático, que altera los eventos meteorológicos, impacta la dinámica ambiental e influye directamente en la ocurrencia, frecuencia y magnitud de fenómenos extremos, como sequías, inundaciones y olas de calor ^2^. Estos eventos, a su vez, impactan en la proliferación de vectores y aumentan la presencia de enfermedades en períodos no previstos o en áreas previamente no afectadas. Tales escenarios requieren el desarrollo de nuevas capacidades para responder, predecir y anticipar eventos inusuales, además de mejorar el abordaje de las complejidades asociadas a distintos perfiles de morbimortalidad. Dichas cuestiones se ven agravadas por gigantescas desigualdades sociales que, al superponer vulnerabilidades, afectan desproporcionadamente a las poblaciones más pobres, negras e indígenas.

En respuesta a estos desafíos, el 12.º Epi incorporó en su estructura un nuevo eje temático, *Emergencias de Salud Pública, Cuestiones Climáticas Mundiales y Modelos de Producción*. Este eje abordó el cambio climático, sus interacciones con el trabajo humano y los procesos productivos y sus efectos en la salud y en las emergencias de salud pública. También cobró énfasis el eje de las *Interfaces con la Sociedad: Información, Educación y Comunicación en Salud*, que resaltó el papel esencial de la difusión y traducción del conocimiento para la defensa de la ciencia y de las acciones respaldadas en la evidencia científica. Los compromisos históricos de la epidemiología brasileña continuaron guiando las actividades con los ejes *Desafíos y Avances Teóricos y Metodológicos*, *Vigilancia en Salud: Innovación, Información y Acción*, *Desafíos en la Formación en Epidemiología*, *Sostenibilidad, Difusión e Integridad en la Investigación*, *Enfoques Innovadores en Estudios Epidemiológicos Centrados en Problemas de Salud y Grupos Poblacionales Específicos* y *Determinación Social de los Procesos Salud-Enfermedad en Contextos de Mayor Vulnerabilidad*. Así, el programa se estructuró en torno a los ocho ejes temáticos contemporáneos del área, explorando las fronteras del conocimiento, identificando futuras necesidades de investigación y políticas públicas capaces de atender esta amplia constelación de intereses.

Entre los múltiples desafíos relacionados con los problemas de salud abordados en estos ejes temáticos, destacó la relevancia y la oportunidad de avanzar en la discusión y en la proposición de acciones para su afrontamiento. Las desigualdades producidas por un modelo económico neoliberal codicioso y concentrador de riqueza, así como sus efectos en la salud, surgieron como temas centrales y temas transversales a lo largo del evento. También ganaron protagonismo las iniciativas encaminadas a combatir el hambre y la pobreza. Los determinantes sociales de la salud fueron ampliamente discutidos en conferencias, ponencias y otras actividades, reforzando el entendimiento de que la producción de conocimiento sobre estas inequidades y sus impactos en la salud es esencial para fortalecer acciones que promuevan el acceso equitativo a políticas públicas y servicios de salud. Además, se priorizaron investigaciones y experiencias orientadas a reducir las desigualdades regionales, económicas, de género y de raza/color, con el objetivo de consolidar una perspectiva de salud verdaderamente colectiva.

Recientemente, Río de Janeiro se convirtió en el centro de las atenciones mundiales al albergar la reunión del Cumbre del G20 (G20). Durante las actividades preparatorias, se resaltaron prioridades estratégicas para la agenda mundial, incluyendo la prevención, preparación y respuesta a pandemias, con énfasis en fortalecer la producción local y regional de medicamentos, vacunas e insumos. Otros puntos incluyeron la salud digital, con el objetivo de ampliar la telesalud, la integración de los sistemas nacionales de salud y el análisis de datos para optimizar los servicios. Las cuestiones de equidad en salud y los impactos del cambio climático en la salud también ganaron protagonismo, reforzando la relevancia de estos temas en la construcción de sistemas de salud más resilientes y sostenibles. Estas prioridades fueron igualmente valorizadas en nuestra programación.

Las crisis sanitarias, como lo demuestra la pandemia de COVID-19, ponen de relieve la necesidad urgente de desarrollar estrategias de respuesta rápida, que incluyan sistemas sólidos de seguimiento y vigilancia, integración de datos y participación de las comunidades locales. Las iniciativas de varios grupos de epidemiólogos brasileños, ampliamente difundidas en las actividades de 12.º Epi, han logrado avances significativos en esta dirección, contribuyendo a fortalecer la capacidad para enfrentar emergencias de salud pública. En un escenario en el que la vacilación vacunal se ve agravada por la desinformación, se reforzó en el congreso el papel de la epidemiología para fortalecer la confianza en las vacunas y en otras intervenciones eficaces de salud pública. También obtuvo protagonismo el debate sobre las innovaciones digitales, como el análisis de *big data* y el uso de la inteligencia artificial, explorando tanto sus potencialidades como los desafíos relacionados con la privacidad y con el uso responsable de datos. La carga de enfermedades crónicas y los problemas de salud mental también fueron ampliamente abordados en diversas actividades, aportando valiosas reflexiones sobre las innovaciones necesarias en vigilancia de la salud, en las estrategias de prevención, en las líneas de cuidado y en otras acciones destinadas a enfrentar esos desafíos en el Sistema Único de Salud (SUS) brasileño.

Para agravar este escenario, las violencias, profundamente arraigadas en nuestra cultura esclavista y sexista, también estuvieron en el centro del debate. El racismo, la violencia institucional, la violencia urbana, la violencia de género, los abusos contra niños y adolescentes, las lesiones autoinfligidas y el suicidio fueron algunos dos temas discutidos. Se reforzó que las violencias no solo se cobran vidas, sino que también causan graves daños a la salud de los supervivientes, de sus familias y de la sociedad en su conjunto. Aunque afectan a gran parte de la población brasileña, sus consecuencias son especialmente devastadoras para los grupos más vulnerables, como mujeres, niños, adolescentes y jóvenes negros pobres, que viven en la periferia.

Desde el I Congreso Brasileño de Epidemiología, celebrado en 1990, en Campinas (São Paulo), hemos sido testigos de cómo la diversidad de temas, el intercambio de experiencias, los aprendizajes, los encuentros y el fortalecimiento del área han sido marcas registradas de los eventos. Estos mismos elementos contribuyeron al gran éxito del 12.º Epi. El congreso reunió alrededor de 3.400 participantes de todas las regiones de Brasil y de otros países. El evento fue más que un encuentro de investigadores(as) y profesionales de la salud; se convirtió en un símbolo del compromiso de Associación Brasileña de Salud Colectiva (Abrasco), especialmente de su Comisión de Epidemiología, con la democracia, el fortalecimiento de la salud colectiva, la defensa del SUS, la valorización de la diversidad y la reducción de las desigualdades. Entre las diversas acciones implementadas en ese sentido a lo largo de la organización del congreso, destaca el compromiso de la Comisión Científica y de la Organización Local con la promoción de la diversidad en sus múltiples dimensiones − temática, institucional, generacional, regional, de género y de raza/etnia/color − en la composición de mesas redondas, ponencias y conferencias. Además, se adoptaron acciones afirmativas para aumentar la participación de poblaciones vulnerables y monitores(as) voluntarios(as), incluida la exención de las tarifas de inscripción y el suministro de comidas. Estas iniciativas, garantizadas por la Comisión Organizadora Local, enriquecieron enormemente las discusiones a lo largo del evento.

El programa del congreso fue amplio y variado. El Precongreso, celebrado en la Universidad del Estado de Río de Janeiro (UERJ), contó con 48 actividades, distribuidas en 25 cursos, 21 talleres y dos reuniones. 12.º Epi, a su vez, contó con siete conferencias, ocho ponencias y 62 mesas redondas, que movilizaron a 234 invitados(as) y 94 moderadores(as). La mayor parte de las actividades se construyó con la participación activa de la comunidad de epidemiólogos de todo el país, por medio de una convocatoria pública abierta para cursos, talleres, mesas redondas y ponencias. Las actividades de comunicaciones coordinadas, pósteres dialogados y pósteres electrónicos reflejaron también la amplia y activa participación de la comunidad, reuniendo trabajos desarrollados en diferentes niveles de formación y actuación, provenientes de todo el País. Se enviaron 3.380 trabajos, de los cuales 2.802 fueron informes de investigación (82,9%) y 579 de experiencia (17,1%). El gran número de resúmenes aprobados resultó en 65 sesiones de comunicación coordinada, que abarcaron 312 trabajos, 60 sesiones de pósteres dialogados con la presentación de 314 trabajos. Entre las comunicaciones coordinadas, 15 trabajos recibieron menciones honoríficas. Estos fueron los temas más abordados: enfermedades transmisibles, enfermedades crónicas no transmisibles, vigilancia epidemiológica y vigilancia en salud, epidemiología nutricional, salud del niño y del adolescente, evaluación de sistemas, políticas, programas, servicios y tecnologías médico-sanitarias, salud del adulto/envejecimiento, salud del trabajador y de la trabajadora, epidemiología en subgrupos poblacionales específicos y accidentes y violencias, entre otros. El número de trabajos sobre cada uno de estos temas estará disponible en las actas del evento, que serán publicadas en breve por Abrasco.

Además del rico programa científico, promovimos tres Encuentros Intergeneracionales, creados para fomentar el diálogo entre las generaciones que contribuyeron a la construcción y consolidación de la Epidemiología en Brasil y jóvenes investigadores(as). Estos encuentros resaltaron la importancia del intercambio de experiencias como herramienta esencial para promover la equidad en la salud. Otra iniciativa innovadora de esta edición del congreso fue la creación de un espacio dedicado a debatir los principales desafíos teóricos, conceptuales y metodológicos enfrentados en el desarrollo de grandes estudios epidemiológicos en Brasil. Abarcando una amplia gama de temas de estudio y áreas de actividad, esta actividad sin precedente incluyó 16 sesiones, que reunieron a 38 invitados(as) y atrajeron a más de un centenar de congresistas. Para complementar el programa, el Comité Científico, en colaboración con Abrasco Livros y Editora Fiocruz, organizó la publicación de 16 libros en diversos ejes temáticos. Esta iniciativa representó un importante estímulo a la producción científica y al avance del conocimiento en el área, con gran potencial para inspirar nuevas investigaciones y acciones transformadoras.

El congreso también fue escenario del lanzamiento del V Plan Maestro para el Desarrollo de la Epidemiología en Brasil (2025-2029), en una Sesión Especial. Este hito marcó la reanudación de los planes maestros quinquenales para la Epidemiología en el país, práctica iniciada en 1989, pero interrumpida desde el 2005. Elaborado a lo largo de un extenso proceso de construcción colectiva que comenzó en el 2022, el V Plan se propone orientar los próximos pasos en el desarrollo y futuro de la Epidemiología en Brasil. Sus directrices apuntan a avanzar en la formación profesional, en la investigación, en la integración de datos y en la implementación de políticas públicas más efectivas, con el objetivo de promover la salud, controlar las enfermedades y reducir las desigualdades sociales, regionales, de género, raza y etnia.

Todas estas actividades reflejaron el vigor del área, su amplitud y diversidad. La construcción de un congreso de tal riqueza solo fue posible gracias a un minucioso trabajo colectivo, apoyado en alianzas, solidaridad, objetivos comunes y un profundo compromiso con la salud colectiva y la epidemiología, todo ello combinado con una dedicación incansable. El Comité Científico, integrado por 36 miembros, desempeñó un papel central, reuniendo esfuerzos de la comisión de epidemiología de Abrasco, investigadores de diferentes instituciones, y de profesionales de diversos departamentos de la Secretaría de Vigilancia de la Salud y del Medio Ambiente del Ministerio de Salud. La Comisión Local, por su parte, contó con la participación de 31 miembros de las instituciones promotoras del Congreso - UERJ, Fundación Oswaldo Cruz (FIOCRUZ), Universidad Federal de Río de Janeiro (UFRJ), Universidad Federal Fluminense (UFF) y Instituto de Educación Médica (IDOMED). La Secretaría de Abrasco estuvo empeñada y dedicada a apoyar las acciones y la Presidencia del Congreso participó activamente en todos estos frentes. 12.º Epi, en toda su magnitud, fue el resultado de esta fuerza colectiva y del empeño conjunto de todos los involucrados.

También nos gustaría resaltar los avances hacia la equidad de género en la epidemiología brasileña. Las mujeres asumieron los puestos de coordinación en la organización y programación: hubo dos presidentas del Congreso, además de dos presidentas de honor, una presidenta de la Comisión Científica y dos coordinadoras de la Comisión Local. El congreso también brindó un momento de reconocimiento a investigadores e investigadoras con contribuciones destacadas al desarrollo de la epidemiología en el país al homenajear a los profesores(as): Ligia Regina Franco Sansigolo Kerr (Universidad Federal de Ceará), Antônio Augusto Moura da Silva (Universidad Federal de Maranhão), Rosely Sichieri (UERJ), Célia Landmann Szwarcwald (FIOCRUZ), César Gomes Victora (Universidad Federal de Pelotas) y José Eluf Neto (Universidad de São Paulo, *in memoriam*).

La ciudad de Río de Janeiro, sede de 12.º Epi, simboliza de manera emblemática la complejidad y los desafíos sanitarios que caracterizan a nuestro país. Por cada playa de Copacabana, se enfrentan las amenazas del narcotráfico y de las milicias; mientras se celebran las emociones en el Maracaná, lidiamos con los problemas de movilidad urbana; y al lado del Cristo Redentor, convivimos con niños en situación de vulnerabilidad. Estos contrastes reflejan no solo la realidad de Río de Janeiro, sino también la de Brasil en su conjunto, y refuerzan la relevancia de reunirse y discutir formas de transformar esta realidad.

Ante tantos desafíos, 12.º Epi reafirmó el poder transformador de una ciencia comprometida, capaz de generar soluciones para construir un futuro más justo y solidario. Una Ciencia que promueve la inclusión social, respeta la diversidad y profundiza nuestra comprensión de los principales problemas de salud que afectan a nuestra población brasileña. Además, busca a aliados(as) de otros países que aporten colaboraciones inspiradoras e innovadoras. Este congreso fue más que un espacio para el intercambio de conocimiento; fue una oportunidad para renovar nuestro compromiso con el fortalecimiento de una ciencia que combata la pobreza, reduzca las desigualdades y oriente políticas que tengan como objetivo la equidad y la justicia social. 12.º Epi fue una respuesta firme al negacionismo científico que marcó al gobierno anterior. Boicotear la ciencia y fomentar la difusión de información falsa contribuyó directamente a cientos de miles de muertes por COVID-19 y otras enfermedades. En este contexto, el congreso reafirmó la relevancia de la ciencia basada en evidencias y su papel esencial en la defensa de la vida y de la salud pública.

Finalmente, nos gustaría reafirmar que durante todo el proceso de construcción del evento buscamos reforzar nuestro compromiso con la Democracia, el SUS, la lucha por una sociedad más justa, menos desigual y libre de discriminación. La plena participación de profesionales de la salud, investigadores(as), estudiantes de pregrado y posgrado fue la base para el debate constructivo y democrático sobre los temas que componen la complejidad sanitaria que tanto nos moviliza. Nosotros(as), de la comisión organizadora del 12.º Epi, esperamos que el congreso haya inspirado nuevas ideas, fortalecido los compromisos con una sociedad más justa y solidaria y renovado las energías para enfrentar los desafíos de la Salud Colectiva.

Motivados por tantos problemas que superar, ¡sigamos adelante!

¡Viva la Ciencia, viva el SUS, viva Abrasco y la Epidemiología brasileña!
